# The blue butterfly Polyommatus (Plebicula) atlanticus (Lepidoptera, Lycaenidae) holds the record of the highest number of chromosomes in the non-polyploid eukaryotic organisms

**DOI:** 10.3897/CompCytogen.v9i4.5760

**Published:** 2015-10-07

**Authors:** Vladimir A. Lukhtanov

**Affiliations:** 1 Department of Karyosystematics, Zoological Institute of Russian Academy of Sciences, Universitetskaya nab. 1, 199034 St. Petersburg, Russia; 2 Department of Entomology, Faculty of Biology, St. Petersburg State University, Universitetskaya nab. 7/9, 199034 St. Petersburg, Russia

**Keywords:** *Acipenser*, *Amoeba proteus*, *Astacus*, *Aulacantha scolymantha*, chromosome number, karyotype evolution, linkage group, Lycaenidae, *Ophioglossum*, *Pacifastacus*, *Plebicula*, *Polyommatus*, vizcacha rat

## Abstract

The blue butterfly species Polyommatus (Plebicula) atlanticus (Elwes, 1906) (Lepidoptera, Lycaenidae) is known to have a very high haploid number of chromosomes (n= *circa* 223). However, this approximate count made by Hugo de Lesse 45 years ago was based on analysis of a single meiotic I metaphase plate, not confirmed by study of diploid chromosome set and not documented by microphotographs. Here I demonstrate that (1) *Polyommatus atlanticus* is a diploid (non-polyploid) species, (2) its meiotic I chromosome complement includes at least 224-226 countable chromosome bodies, and (3) all (or nearly all) chromosome elements in meiotic I karyotype are represented by bivalents. I also provide the first data on the diploid karyotype and estimate the diploid chromosome number as 2n=ca448-452. Thus, *Polyommatus atlanticus* is confirmed to possess the highest chromosome number among all the non-polyploid eukaryotic organisms.

## Introduction

Trends and mechanisms of chromosome number and chromosome structure changes are currently a matter of a sharp discussion (Qumslyeh 1994, Imai et al. 2002, Eichler and Sankoff 2003, Schubert 2007, Lukhtanov et al. 2005, 2011, 2015a, Vila et al. 2010, Dincă et al. 2011, Bureš and Zedek 2014, Fleischmann et al. 2014, Lukhtanov 2014, Vershinina et al. 2015). These changes are important in evolution of eukaryotic organisms since they can trigger speciation via hybrid-sterility or/and via suppressed-recombination mechanisms (Faria and Navarro 2010). Fixation of these changes plays a serious role in maintaining postzygotic isolation between well-established species and protects hybridizing lineages from merging (Kandul et al. 2007). Change of chromosome number results in change of linkage groups and thus affects rate of meiotic recombination (Dumont and Payseur 2011).

Comparative analysis of chromosomal data is a promising way for understanding the patterns of karyotype evolution (Vershinina and Lukhtanov 2013), and this analysis requires accurate and precise data on chromosome complements of species under study. The blue butterfly Polyommatus (Plebicula) atlanticus (Elwes, 1906) is mentioned in many publications devoted to chromosome number evolution since it is supposed to possess the highest chromosome number (n= *circa* 223) among all the non-polyploid metazoan animals (e.g. White 1973, Imai et al. 2002, Bureš and Zedek 2014). However, this approximate count made by Hugo de Lesse 45 years ago was based on analysis of a single meiotic I metaphase plate, not confirmed by studies of diploid chromosome set and not documented by microphotographs (de Lesse 1970).

The aim of this study is cytogenetic reinvestigation and documentation of *Polyommatus atlanticus* karyotype with a special consideration of diploid chromosome set of this species.

## Material and methods

The studied species is often mentioned in the literature as a member of the genus *Lysandra* Hemming, 1933 (e.g. de Lesse 1970, White 1973). However, according to the last revision of the tribe Polyommatina, it should be transferred to the genus *Polyommatus* Latreille, 1804 (Talavera et al. 2013a). The adult male samples used for chromosomal analysis (NK02A032, NK02A033 and NK02A035) were collected in Morocco (Atlas range, Col du Zad pass, 2200 m alt., 27 June 2002) by Roger Vila, Santiago Ramirez and Nikolai Kandul. The methods of chromosomal analysis were described previously (Lukhtanov and Dantchenko 2002, Lukhtanov et al. 2008, 2014, Vershinina and Lukhtanov 2010, Talavera et al. 2013b, Przybyłowicz et al. 2014). Haploid (n) chromosome numbers were analyzed in meiotic I (MI) and meiotic II (MII) cells. Diploid (2n) chromosome numbers were analyzed in asynaptic meiotic cells that can be observed in so called atypical meiosis (see Lorković 1990 for more details on atypical meiosis in Lepidoptera).

## Results

**Figure 1.**
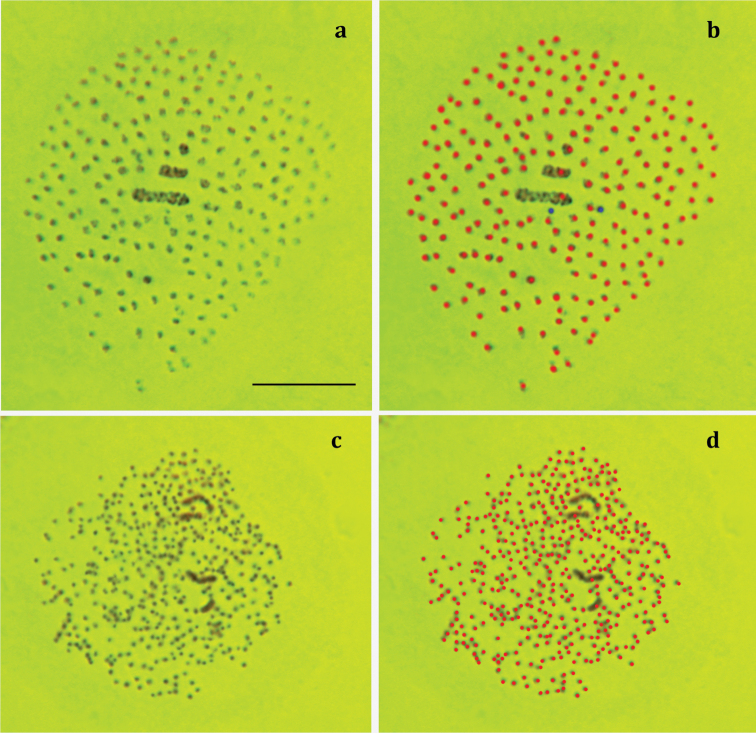
Male karyotype of Polyommatus (Plebicula) atlanticus, sample NK02A032. **a** MI plate **b** chromosome count in MI plate: red dots indicate distinct separate entities, blue dots indicate doubtful entities, n=224 red dots + 2 blue dots **c** diploid chromosome set observed in male asynaptic meiosis **d** chromosome number count in diploid chromosome set; at least 434 entities can be distinguished. Bar = 10 μm.

The haploid chromosome number n=ca 224–226 was found in MI cells of three studied individuals (Fig. 1a, b). This count was based on analysis of 12 selected MI plates with best quality of chromosome spreading. The meiotic karyotype included one large bivalent, one medium bivalent and 222–224 small chromosome bodies. Multiple MII cells were also observed. The MII cells demonstrated one large and one medium chromosome and multiple dot-like elements, however the precise count of these elements was impossible. The diploid chromosome set was observed in male atypical (asynaptic) meiosis (Fig. 1c, d) in three studied individuals (20 cells were analysed). At this stage at least 434 chromosome entities could be observed: one pair of large chromosomes, one pair of medium chromosomes and at least 430 (most likely more) very small, dot-like chromosomes. Combination of chromosome number count at MI and diploid stages results in conclusion that all (or nearly all) chromosome elements in MI karyotype are represented by bivalents. This assumption results in diploid chromosome number estimation of 2n=ca 448–452.

## Discussion

Previously, the chromosome number was estimated in *Polyommatus atlanticus* as n=ca217-223 (de Lesse 1970). This number has later been interpreted as 2n=446 (e.g. see Bureš and Zedek 2014). However, interpretation of all chromosome bodies visible at MI stage as bivalents should be considered with caution. As it was mentioned by White (1973), “there seems to be no means of distinguishing between univalents, bivalents and multivalents in lepidopteran spermatogenesis – they all look like small spheres or isodiametric bodies in which no structure is observable”. For example, multiple B-chromosomes (which can be often represented by univalents in meiosis) can sometimes accumulate through processes of mitotic or meiotic drive (Jones 2008). Therefore, I believe that analysis of diploid karyotype is indispensable prerequisite for inferring the diploid chromosome number. In my research the combination of chromosome number counts at MI and diploid stages results in conclusion that all (or nearly all) chromosome elements in MI karyotype are represented by bivalents. This assumption leads to conclusion that diploid chromosome number can be estimated in *Polyommatus atlanticus* as 2n=ca 448–452, and the haploid number can be estimated as n=ca 224–226.

In eukaryotic organisms the highest number of chromosomes has been so far reported in radiolarian species, e.g. in *Aulacantha scolymantha* Haeckel, 1862 (Cercozoa, Aulacanthidae) there are more than 2000 chromosomes (Lecher 1978). This high number is an output of polyploidization (Lecher 1978, Parfrey et al. 2008), which includes 7 or 8 cycles of endomitosis resulting in each chromosome represented by 128 or 256 copies (Lecher 1978).

500 chromosomes were reported for asexual lobose amoebae, *Amoeba proteus* (Pallas, 1766) (Amoebozoa, Amoebidae) (Parfrey et al. 2008). This high number is also considered to be polyploid although the questions about the precise number of chromosomes and the ploidy level are still unanswered despite the fact that cytology of this well-known species has been under study for about 200 years (Podlipaeva et al. 2013).

Very high chromosome numbers are known in some plants, e.g. in ferns of the genus *Ophioglossum* Linnaeus, 1753 (Pteridophyta, Ophioglossaceae) n=120–720 (Shinohara et al. 2013). However, this genus is also characterized by a high degree of polyploidization with x=120 as a basic chromosome number and with the highest n=720 in hexaploid species *Ophioglossum reticulatum* Linnaeus, 1753 (Khandelwal 1990, Barker 2013, Shinohara et al. 2013).

In vertebrate animals the highest chromosome number (372 elements in mitotic cell divisions) is known in sturgeon *Acipenser brevirostrum* Lesueur, 1818 (Acipenseriformes, Acipenseridae) (Kim et al. 2005), however this species is hexaploid one, too (Kim et al. 2005). In mammals the highest chromosome number 2n=102 is found in vizcacha rat *Tympanoctomys barrerae* (B. Lawrence, 1941) (Rodentia, Octodontidae) (Suárez-Villota et al. 2012).

According to White (1973), the highest haploid chromosome number recorded in invertebrate animals (except for *Polyommatus atlanticus*) is n=191 in the butterfly *Polyommatus nivescens* (Keferstein, 1851) (Lepidoptera, Lycaenidae) (de Lesse 1970, White 1973). The next highest haploid numbers were reported in crayfish, *Pacifastacus leniusculus trowbridgii* (Stimpson, 1857) (Crustacea, Astacidae) (n=188, Niiyama 1962) and *Astacus leptodactylus* (Eschscholtz, 1823) (Crustacea, Astacidae) (n=184, Silver and Tsukersis 1964). The last two counts were even erroneously cited as the records for the highest chromosome numbers in the animal kingdom (Fetzner and Crandall 2002). However, the numbers in crayfish are, first, lower than the numbers discovered in the blue butterflies. Second, they were disputed in the more recent publications (e.g. n=93 was mentioned in *Pacifastacus leniusculus trowbridgii*, Murofushi 1999, Imai et al. 2002 and n=90 was mentioned in *Astacus leptodactylus*, Mlinarec et al. 2011). All these haploid numbers are essentially lower than numbers found in *Polyommatus atlanticus*.

The data obtained indicate that *Polyommatus atlanticus* is a diploid (not polyploid) species since it possesses double (not multiple) number of chromosomes that can be individually recognized: one pair of large and one pair of medium chromosomes. Thus, *Polyommatus atlanticus* is confirmed to have the highest chromosome number among all the non-polyploid eukaryotic organisms.
